# Supplementary figures and images for: Global Composition of the Bacteriophage Community in Honey Bees
Source: mSystems. 2022 Mar 28;7(2):e01195-21. doi: 10.1128/msystems.01195-21 (PMC9040601; doi:10.1128/msystems.01195-21)

Genome Quality

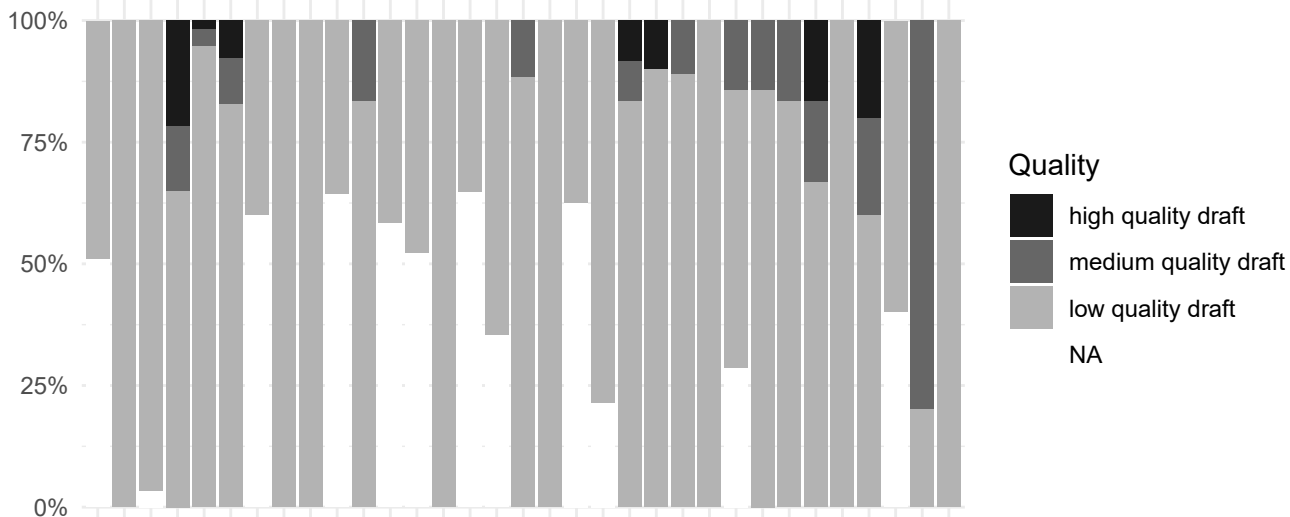

Circular

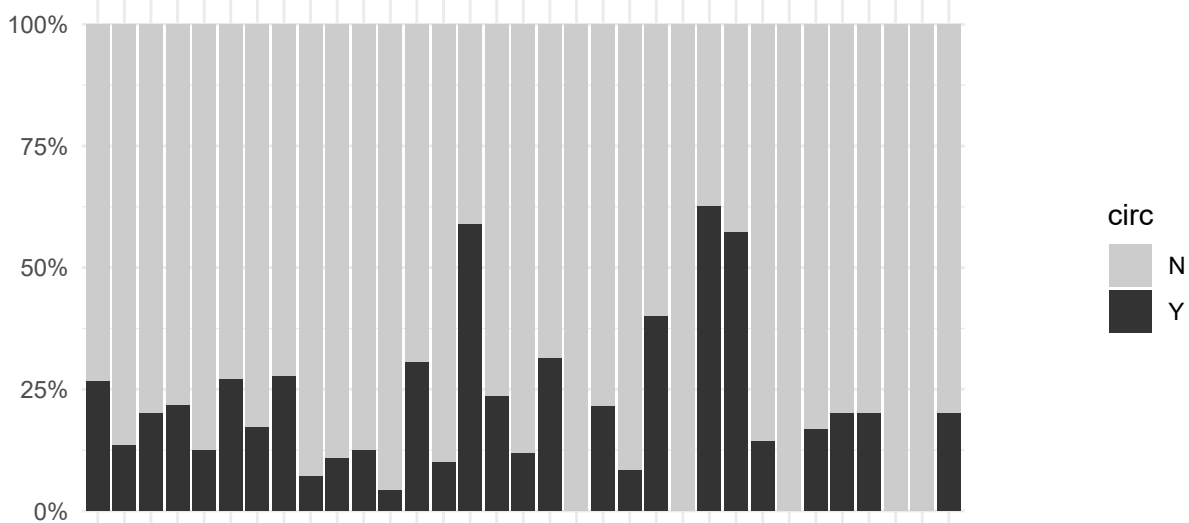

Intersection size

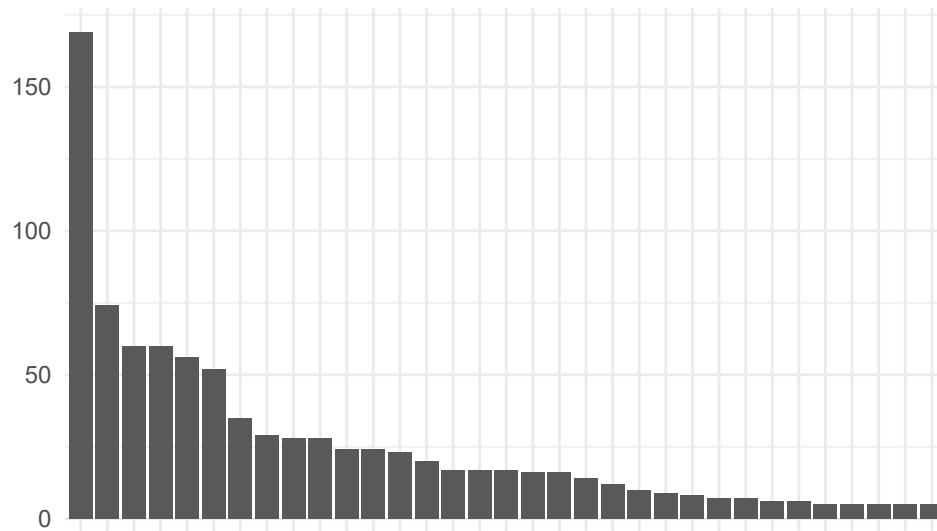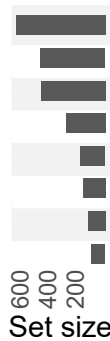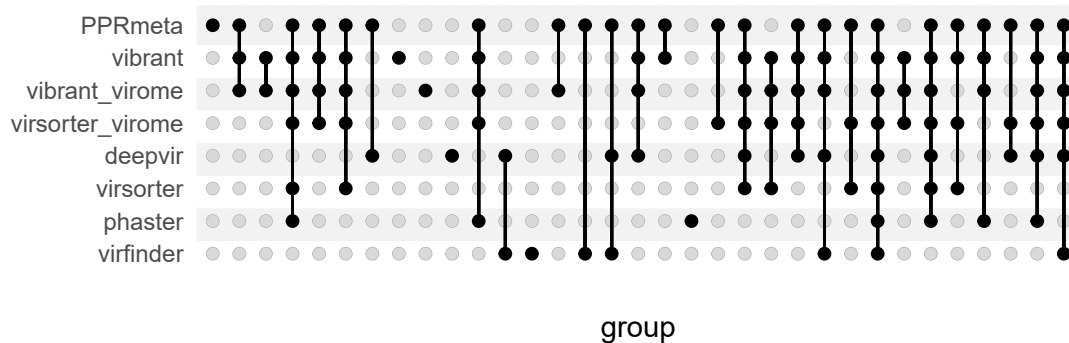

Supplement: FIG S1 [file msystems.01195-21-sf001.pdf]

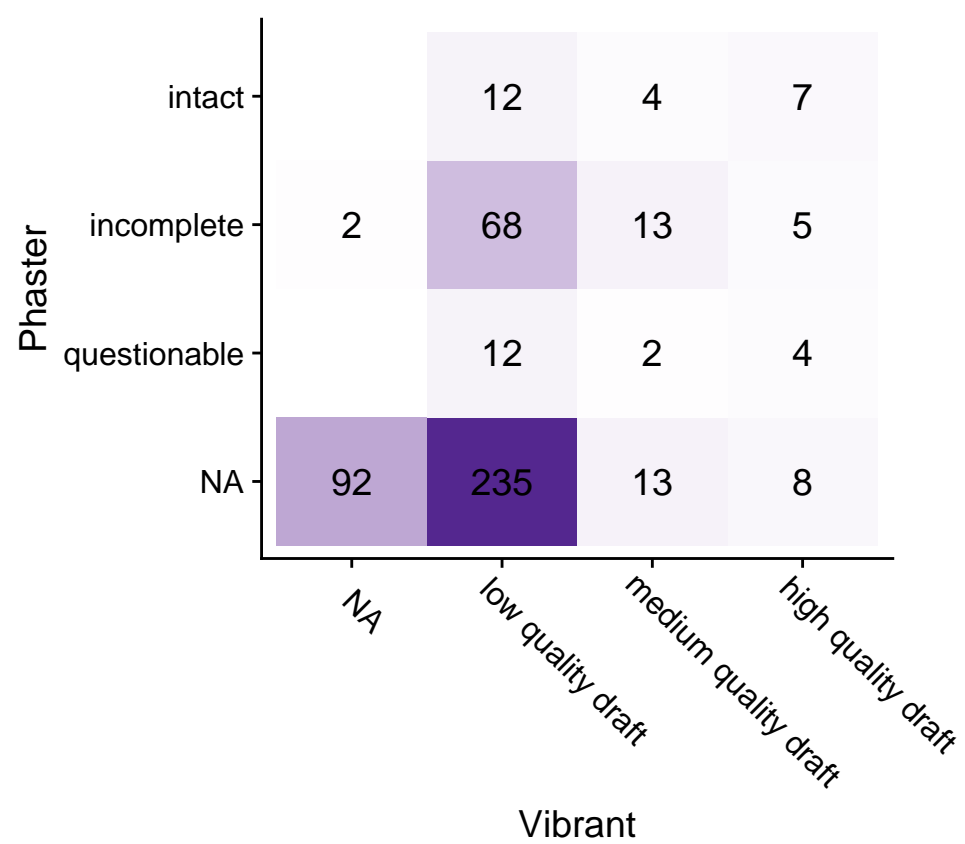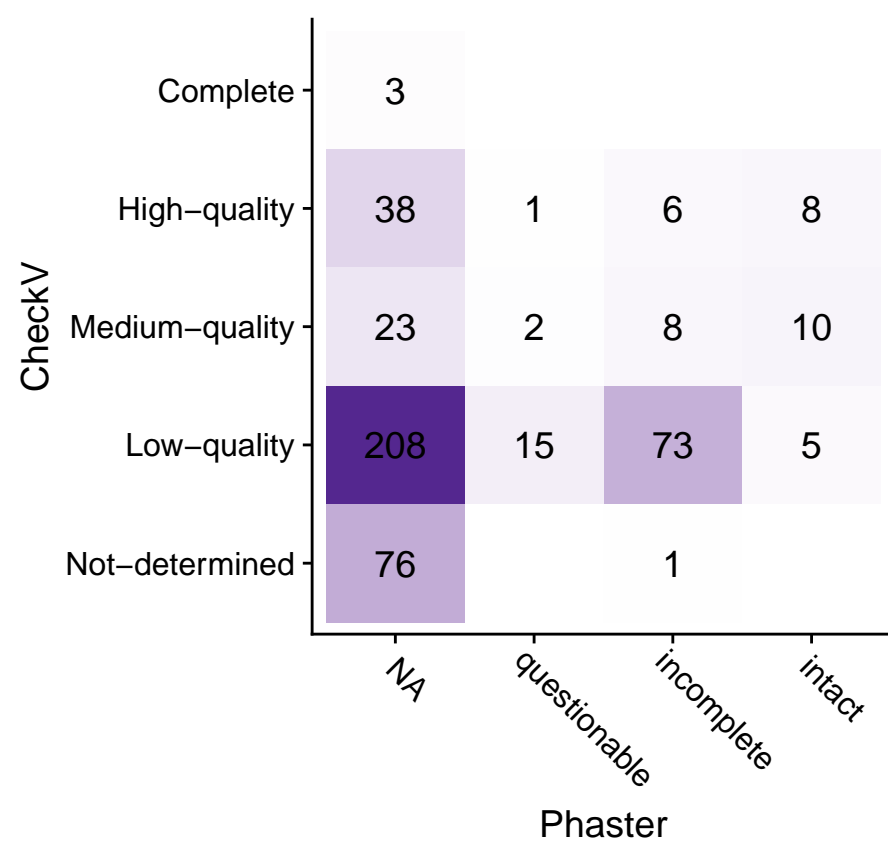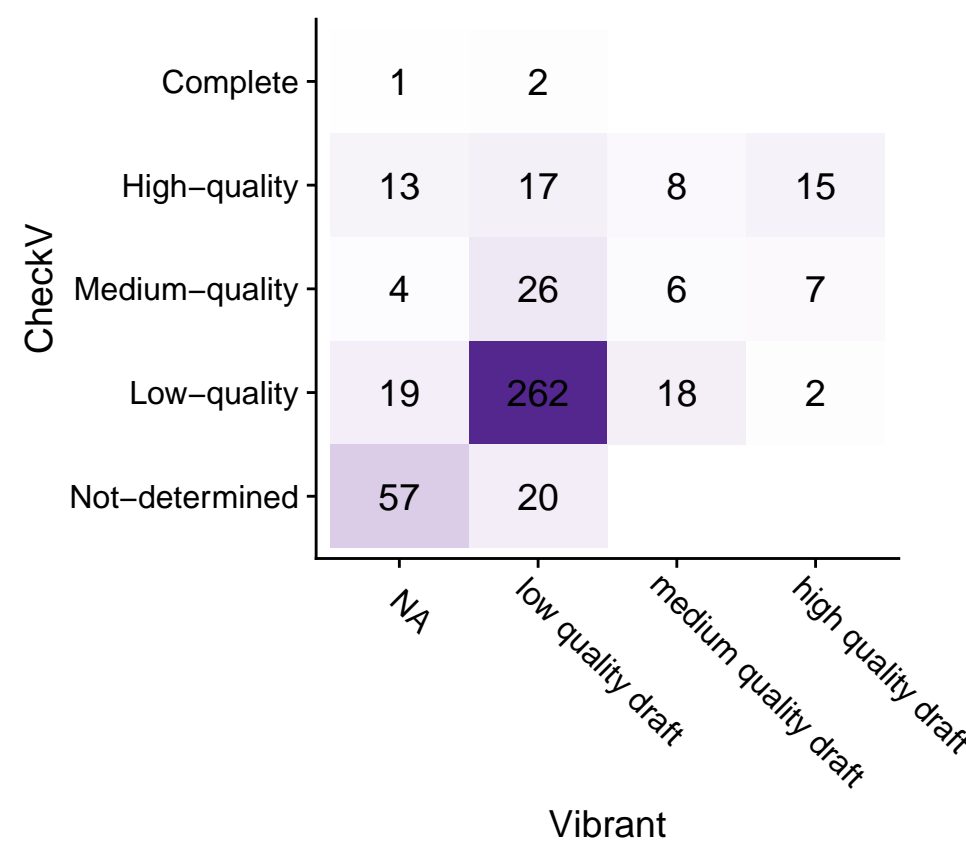

Supplement: FIG S2 [file msystems.01195-21-sf002.pdf]

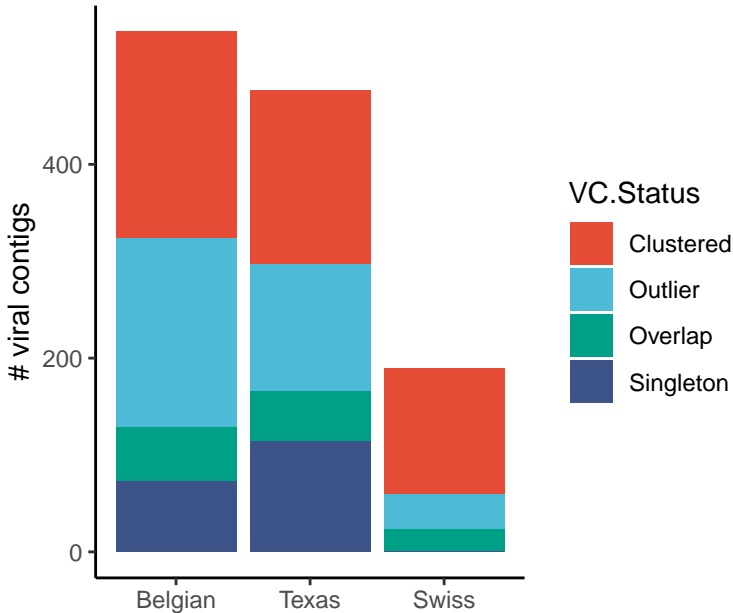

Supplement: FIG S3 [file msystems.01195-21-sf003.pdf]

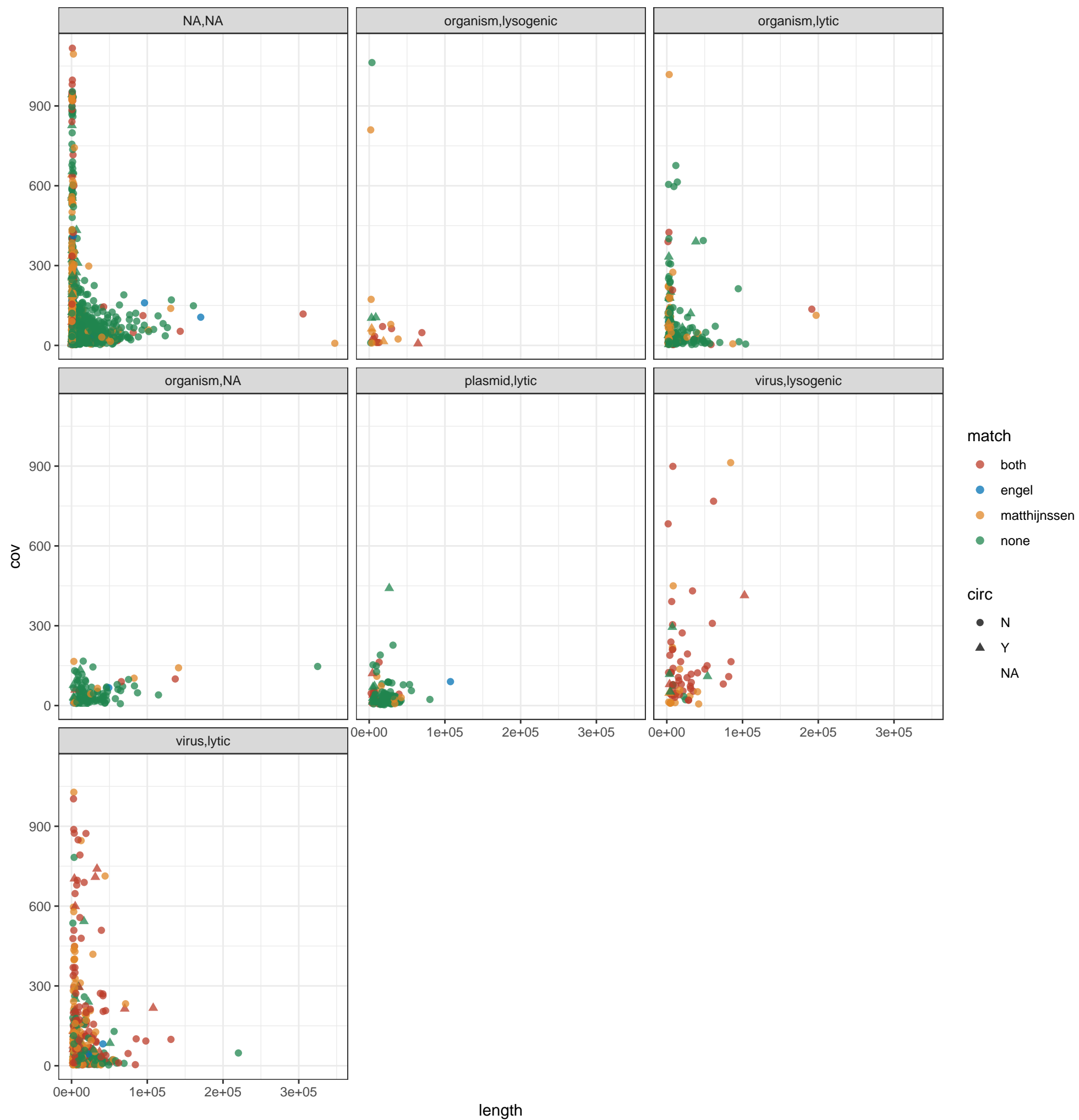

Supplement: FIG S5 [file msystems.01195-21-sf005.pdf]

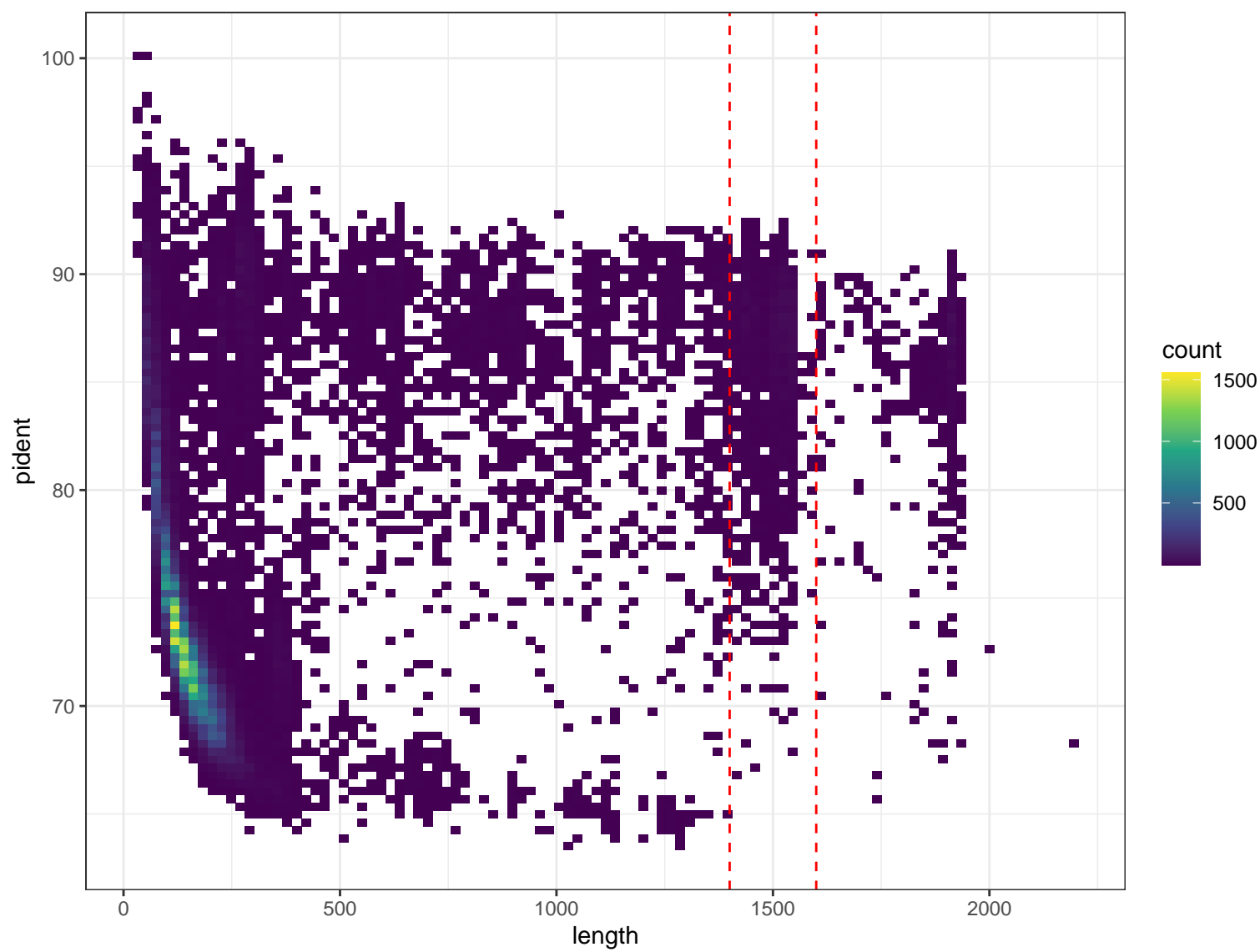

Supplement: FIG S4 [file msystems.01195-21-sf004.pdf]
